# Implementing guidelines on physical health in the acute mental health setting: a quality improvement approach

**DOI:** 10.1186/s13033-018-0179-1

**Published:** 2018-01-10

**Authors:** Stuart Green, Ed Beveridge, Liz Evans, Jenny Trite, Sandra Jayacodi, Rachel Evered, Caroline Parker, Luca Polledri, Emily Tabb, John Green, Anton Manickam, Joanna Williams, Rebecca Deere, Bill Tiplady

**Affiliations:** 1 NIHR CLAHRC Northwest London, Imperial College London, Chelsea and Westminster Hospital, London, UK; 2 St Charles Mental Health Centre, Central and North West London NHS Foundation Trust, London, UK; 3 Latimer House, Central and North West London NHS Foundation Trust, London, UK

**Keywords:** Clinical practice guideline, Quality improvement, Mental health services, Healthcare inequalities

## Abstract

**Background:**

In the UK, life expectancy for people living with a serious mental illness, such as schizophrenia and bipolar disorder, is reduced by 15–20 years compared with the general population. In recent years, evidence based guidelines/policies designed to improve their physical health have been published, yet a gap remains between recommendations and practice. This case study describes how guidelines to support physical health were implemented using a quality improvement approach.

**Case presentation:**

A quasi-experimental study explored systems and processes for assessing the physical health of patients admitted to an acute mental health unit. The multi-disciplinary team of healthcare professionals, service users and experts in quality improvement methods developed solutions to improve the assessment of physical health, drawing on existing guidelines/policies as well as professional and lived experience. Three key interventions were developed: a comprehensive physical health assessment; a patient-held physical health booklet; and education and training for staff and patients. Interventions were co-designed by front-line healthcare staff and service users with iterative development and implementation through Plan-Do-Study-Act cycles. Real-time weekly data were reported on five measures over a 15-month implementation period (318 patients) and compared to a 10-month baseline period (247 patients) to gauge the success of the implementation of the physical health assessment. Improvements were seen in the numbers of patients receiving a physical health assessment: 81.3% (201/247) vs 96.9% (308/318), recording of body mass index: 21.55% (53/247) vs 58.6% (204/318) and systolic blood pressure: 22.35% (55/247) vs 75.9% (239/318) but a reduction in the recording of smoking status: 80.1% (198/247) vs 70.9% (225/318). However, 31.7% (118/318) patients had a cardiovascular risk-score documented in the implementation phase, compared to none in the baseline.

**Conclusion:**

This study demonstrates the use of a quality improvement approach to support teams to implement guidelines on physical health in the acute mental health setting. Reflections of the team have identified the need for resources, training, support and leadership to support changes to the way care is delivered. Furthermore, collaborations between service users and frontline clinical staff can co-design interventions to support improvements and raise awareness of the physical health needs of this population.

## Background

In the UK, life expectancy for people living with a serious mental illness (SMI), such as schizophrenia and bipolar disorder, is reduced by 15–20 years compared with the general population [1, 2]. In addition there is often a higher prevalence of physical health disorders that are attributed to increased mortality, for example mortality due to diseases of the respiratory system (4 times greater than the general population) and the circulatory system (2.5 greater than times the general population) [3]. Whilst the true nature of the underlying cause resulting in the increased likelihood of developing physical health conditions leading to higher mortality is not yet fully understood there are undoubtedly links to modifiable factors, such as environment and lifestyle; for example high levels of tobacco consumption, poor diet and reduced physical exercise leading to obesity, which itself is often linked to iatrogenic weight gain associated with antipsychotics [4]. This is further compounded by “diagnostic overshadowing”, when health professionals fail to take people with mental illness seriously when they raise concerns about their physical health, and the systemic failure to assess, monitor and appropriately manage the physical health of people with a SMI [5, 6].

### Problem description

In an attempt to improve the physical health care of people with SMI and close the life expectancy gap a number of evidence based clinical guidelines and policies have been published over the past decade [7, 8]. Unfortunately there remains significant variation in the implementation of these guidelines and recommendations in mental health care services, as outlined by the National Audit of Schizophrenia (NAS) [9]. As such, the difficulties of implementing policy and guidelines in practice are well known in health care [10]. Assessing the physical health of patients when they are in hospital offers an opportunity to identify risk factors for developing conditions e.g. cardiovascular disease (CVD) or diabetes and provide advice and support on services that can be accessed on discharge. In 2014, a national clinical audit identified that the monitoring of physical health indicators of people with mental health problems within the hospital setting was poor, with only 16% of patients locally receiving regular monitoring of key metabolic factors [9].

### Specific aims

To improve assessment and monitoring an initiative was established that adopted an evidence based approach to quality improvement (QI) to test whether this would support the implementation of guidelines to improve physical health in an acute mental health setting.

## Case presentation

### The treatment setting

The QI initiative was developed within an acute admission ward in an acute mental health unit in a hospital in North-West London. The ward admits approximately 300 patients a year, has 16 beds and 22 clinical staff. During the initiative, there was significant organisational planning to implement a new health information technology system across acute and community services, although this didn’t actually occur during the study period.

### Available knowledge

Existing clinical guidelines on physical health monitoring of people with SMI recommend that patients have their physical health monitored through robust assessment and documenting in the patients’ care records [7, 8]. Physical health monitoring includes:

- Weight or body mass index (BMI), diet, nutritional status and level of physical activity.
- Cardiovascular status, including pulse and blood pressure.
- Metabolic status, including fasting blood glucose, glycosylated haemoglobin (HbA1c) and blood lipid profile.
- Liver, renal and thyroid function, and calcium levels, for people taking long‑term lithium.

Furthermore, clinical guidelines on improving the service user experience in mental health state that people using mental health services should be involved in the planning and delivery of training [11].

### Study design

This quasi-experimental prospective study was developed with a QI approach and supported by Collaboration for Leadership in Applied Health Research and Care Northwest London (CLAHRC NWL), an applied health research programme. CLAHRC NWL is funded by the National Institute of Health Research (NIHR) to develop effective ways to translate research knowledge and evidence into practice. To this end CLAHRC NWL have developed a systematic approach using a range of QI methods. This approach also provides support for front-line staff and patients and members of the public involved in QI initiatives through a range of collaborative activities including coaching, facilitated-workshops and peer-learning events.

The initiative was undertaken over a 20-month period between October 2014 and May 2016 on an acute admission ward. This report combines a practical account of the implementation activities, following the Standards for Quality Improvement Reporting Excellence (SQUIRE 2.0) framework, with some reflection of the more theoretical components [12]. This work builds on previous published studies in the area of implementing guidelines in mental health settings but offers QI perspective [13].

#### Creating a representative improvement team

The multi-professional improvement team was established by a senior psychologist and psychiatrist, the co-leads for the initiative, to include staff from nursing, pharmacy, therapies staff including a fitness trainer, senior management, service user representatives and QI expertise from CLAHRC NWL. Involving those most likely to be affected by any changes was necessary to ensure both the acceptability of any change made and allow all key stakeholders to contribute to the project through the design and testing of interventions. Involvement of service users was guided through the 4PI Framework, developed by the National Survivor User Network to support the involvement of patients and members of the public [14]. The use of this framework to guide service user involvement in this initiative is described in detail elsewhere [15].

#### Developing a shared aim and programme theory

The shared aim and programme theory for the initiative were developed in a facilitated action and effect method (AEM) workshop, which included the improvement team and representatives from the wider clinical team, such as ward staff and managers [16]. The AEM workshop provided an opportunity for the broad range of stakeholders involved in delivering and receiving services in the unit to articulate their perspectives of current problems related to physical health and identify potential solutions that could be tested locally. Having service users as integral members of the improvement team during the development of the action effect diagram (AED) had a significant impact on the programme theory and potential solutions generated including the strong emphasis on involving patients in their physical health and interventions to reduce health risks. As a dynamic “live” document, the AED was updated following subsequent exploratory activities, with information about systems and processes informing the programme theory and resulting in the emergence of additional ideas for potential solutions linked to the aim.

#### Exploring current systems and processes

A facilitated process mapping session was used to describe, in detail, current systems and processes for assessing physical health for newly admitted patients to an acute mental health ward [17, 18]. The process mapping revealed two previously unlinked parallel processes for assessing the physical health of new admissions on the ward, one completed by medical staff, the other by nursing staff. Both processes collected similar data that was recorded in different places on the electronic patient record, none of which was communicated to the patient. Neither the ward nurses nor doctors were previously aware of the others’ work, nor did these processes allow the opportunity for wider multi-disciplinary input e.g. pharmacists or therapists. Other processes were identified that had an impact on the assessment of physical health such as the reporting of blood test results, but this was deemed beyond the current scope of influence of the initiative as the team were only able to address problems on the ward rather than wider organisational issues.

#### Reviewing existing policies, evidence and guidelines

Policies and guidelines were identified that were already promoted both locally and nationally, which included the assessment of physical health within the inpatient setting [7, 8, 19]. These provided a robust evidence and policy base to inform the improvement work. The organisation had also recently been inspected by care quality commission (CQC), a government regulator, who had identified the provision of physical health assessments as a key area for improvement.

#### Assessing baseline data

A local audit was undertaken to quantify the existing levels of physical health assessment to establish whether the parallel processes discovered in the process mapping were effective or not. The retrospective audit over a 10-month period included 247 consecutive adult admissions to the ward, highlighting deficits in the current physical assessment process, as was anticipated: Percentage of patients receiving a physical health assessment—81.3% (201/247); recording of BMI—21.55% (53/247), systolic blood pressure—22.35% (55/247) and smoking status—80.1% (198/247). The evidence base and audit data subsequently informed the development of improvement measures and formed a base line for these measures.

#### Designing interventions

The AED, process map and data from the local audit and review of policies helped the team prioritise areas for improvement that were predicted to have the greatest impact on key factors to achieve the shared aim. Three key interventions were identified that would focus on developing a physical health pathway to support staff to undertake physical health care assessments and recording (intervention 1); support communication about risk and shared decision making (intervention 2); and improve understanding about physical health conditions, their risk factors, assessment of risk and appropriate interventions to reduce risks for both staff and patients (intervention 3). All interventions were co-designed by front-line healthcare staff and service users with iterative development and implementation through Plan-Do-Study-Act (PDSA) cycles to allow small scale feasibility testing in the clinical setting to ensure a fit with local systems and processes [20].

#### Intervention 1: a multi-professional physical health assessment

The improvement team worked with ward staff, including nurses, junior doctors and managers, to review the existing parallel assessment and recording processes carried out by doctors and nurses. Through discussions and consensus the group identified how these could be integrated into a unified process within the clerking system, supported by the development of a new single physical health assessment form including CVD and diabetes risk score, explicitly aligned to existing tools and guidelines, including the Lester Tool and National Institute of Health and Care Excellence (NICE) guidelines [7, 8, 21]. In addition, the form would encourage further input and support from a wider multi-disciplinary team, including pharmacists and therapists. The intervention would also need to be practical, easy to use and acceptable to both patients and staff and as such should be co-designed with frontline staff with input from service users. Using the PDSA framework a series of small tests of change were initiated with the ward staff to develop the content and structure of the assessment iteratively. The JBS3 score was introduced as an evidence-based assessment of a patient’s CVD risk: this provided a mechanism for identifying modifiable risk factors that could inform shared-decision making discussions between clinician and service users about appropriate and acceptable risk-reduction strategies [22].

#### Intervention 2: a patient-held physical health plan

Concurrent with the development and implementation of the physical health assessment, work proceeded to co-design a patient-held physical health plan led by service users. The physical health plan was designed as a patient-held record of their own physical health following assessment. It included parameters such as blood pressure, BMI, smoking status etc. and an estimate of the 10 years and lifetime risk of CVD [22]. The plan was intended to support healthcare professionals and patients discuss the results of the assessment and facilitate shared decision making at discharge about access to appropriate support services (e.g. health trainers, smoking cessation etc.). This was provided along with a physical health advice booklet designed specifically for use by mental health patients from the British Heart Foundation and a completed patient-held medications record [23]. The first drafts of the physical health plan were based on several existing information sources developed by healthcare or voluntary organizations and charities for community physical health to patients. These were tested with patients on the ward and elements were selected and refined using further PDSA cycles until agreement was reached on the achievement of the original objectives. The resulting physical health plan was tailored to allow the personalised recording of the patient’s physical health assessment with any identified risks highlighted.

#### Intervention 3: educational resources to support patients and staff

Ensuring physical health assessments are complete and the information is provided to patients, is a first-step to improving physical health, but without access to interventions and further support patients are unlikely to achieve this goal alone. The “Recovery College” model, which is widely used in the UK, supports self-management through service user education, often addressing broader physical health issues [24]. As part of the initiative, courses for patients in the community and post discharge were co-produced with service users to raise awareness of the importance of good physical health care, how to maintain it and where to get additional support. These face to face courses also provided an opportunity to integrate physical and mental health self- care within the Recovery College curriculum. In addition, courses were available to staff to build their confidence and competence in delivering elements of the physical health assessment and providing information to patients.

#### Data to drive improvements

A number of key improvement measures were identified, as recommended by the Institute for Healthcare Improvement ‘Model for Improvement’ [25]. The measures corresponded to many indicators assessed in the audit, providing a baseline and intervention period for analysis. Four improvement measures allowed continuous analysis across the baseline and intervention periods, whilst the fifth improvement measure was aligned to the introduction of the new requirement to provide individualised risk scores for diabetes and CVD using the Q-Risk and JBS-3 calculators, respectively [22, 26]. Data for improvement measures were collected weekly using the Web Improvement Support for Healthcare (WISH) and provided weekly run charts [27]. Whilst this data would ordinarily be subject to analysis by statistical process control (SPC) to identify changes in processes, the small denominator prevented this approach [28]. Nonetheless, the improvement measures were designed to provide a mechanism for the continuous monitoring of the percentage of patients that received a physical health assessment with the recording of specific parameters as proxies for a complete physical health assessment:

1. Percentage of patients who received a physical health assessment.
2. Percentage of patients for whom the smoking status was documented in the physical health assessment.
3. Percentage of patients for whom the BMI was documented in the physical health assessment.
4. Percentage of patients for whom systolic blood pressure was documented in the physical health assessment.
5. Percentage of patients for whom CVD risk score (JBS-3) was documented in the physical health assessment.

#### Delivering interventions

**Fig. 1:**
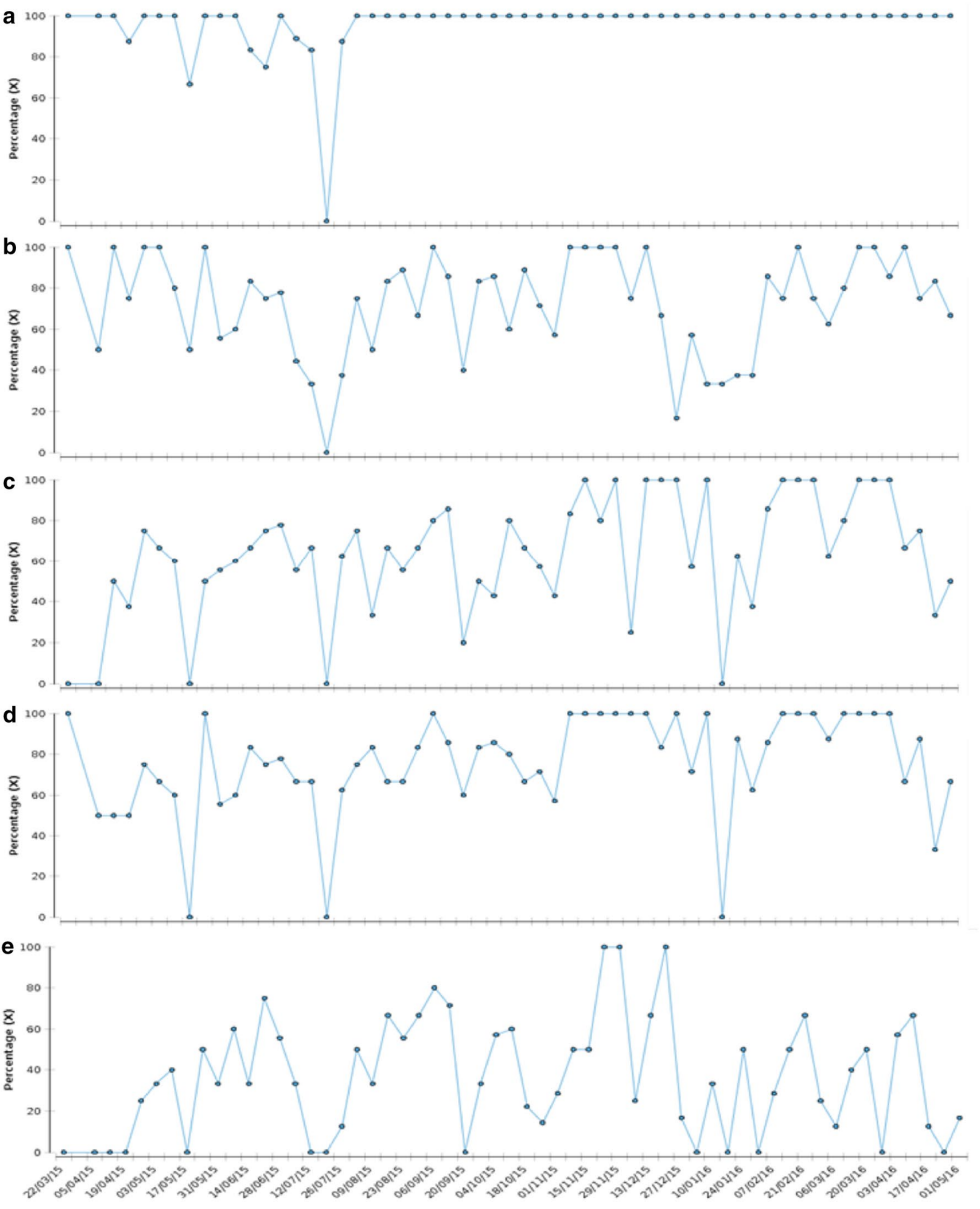
Weekly percentage of patients discharged/transferred from admission ward with a: physical health assessment (**a**); documented smoking status (**b**); documented body mass index (**c**); documented systolic blood pressure (**d**); documented cardio-vascular disease risk score (**e**)

Having iteratively designed the interventions using the PDSA approach the improvement team introduced the physical health assessment to the admission ward through several key strategies, including establishing a ward champion for physical health, one-to-one coaching and educational sessions for ward staff. The initial educational session delivered to the ward (month 3) outlined the current problem relating to the early mortality of people with SMI, and reflected on the Trusts own performance in this area, as assessed by NAS and CQC inspection, and an overview of the interventions introduced to address this problem locally. The improvement team and ward staff were offered training on QI, focussed on the concept of Measuring for Improvement and how to work with PDSA cycles. Following this educational session, the new physical health assessment was introduced to the ward and data collected weekly to monitor implementation and uptake using the five improvement measures. Data were collected by a ward nurse and entered on the WISH tool and time series generated. Visualisation of weekly data through run charts were provided to the improvement team and ward champion each month (Fig. 1a–e). This was supplemented by data about documented reasons where the physical health assessment was incomplete, as a mechanism for collecting qualitative data about challenges in implementing the assessment that could be used to inform modifications to the process. Analysis of the improvement data with the audit data indicate the comparative improvements seen in each of the parameters (Table 1).

**Table 1:** Comparison of the recording of key physical health indicators during the baseline and implementation periods

Indicator	Baseline period n/N (%)	Implementation period n/N (%)
Percentage of patients that received a physical health assessment (PHA)	201/247 (81.3%)	308/318 (96.9%)
Percentage of patients where the smoking status was documented in the PHA	198/247 (80.1%)	225/318 (70.9%)
Percentage of patients where the BMI was documented in the PHA	53/247 (21.6%)	204/318 (58.6%)
Percentage of patients where systolic blood pressure was documented in the PHA	55/247 (22.4%)	239/318 (75.9%)
Percentage of patients where a cardiovascular risk-score was documented in the PHA	N/A	118/318 (31.7%)

## Discussion

The analysis demonstrates improvements in the proportions of patients receiving physical health assessment when comparing the baseline with the average during the study. However, despite the general improvement there is still inconsistency in the delivery of different elements of the assessment, especially the calculation of the CVD risk score. Additionally, the study demonstrates the feasibility of reporting weekly measures, although further work is required to assess the ability to generate this in real-time, which would provide an opportunity to investigate when rates are low and identify local barriers to completion and pro-actively support staff to suggest potential solutions to overcoming these challenges. Moreover, the initiative demonstrates the role that evidence and policies can play in developing interventions for a complex health system, when tailored to the local context, achieved through engaging staff and patients.

### Implementing evidence through local solutions

The design, implementation and evaluation of clinical interventions are often undertaken within a research setting, making their translation to clinical settings contentious at best and impossible at worst [29]. Developing interventions de novo within a clinical setting requires a balance between drawing on existing evidence and alignment to policy, whilst remaining pragmatic and acceptable to the clinical workforce and patients.

Demonstrating a need for change is a powerful starting point for any change initiative, and acts as an organisational and clinical driver for improvement [30]. The baseline audit undertaken by the improvement team demonstrated variation in the delivery of existing care processes, corroborating findings with local external inspections and national audits. In addition to quantifying the ‘opportunity for improvement’ the baseline data provided a platform on which to develop specific measures of improvement. The measures were used by the clinical and improvement teams to review the impact of the implementation of the interventions and provide opportunities to regularly reflect and learn. As such, ‘measurement for improvement’, as a QI method, was the approach most consistently used by the improvement team, with weekly data continuously collected for more than 12-months, and reports generated and reviewed monthly by the improvement team and disseminated to the ward.

Ensuring that interventions are evidence-based and acceptable to both those that deliver them and receive them is a key challenge to implementation. Drawing on existing policies and guidelines, combined with clinical experience and knowledge, provided the improvement team with an opportunity to develop a range of interventions that could support physical health. The co-design of interventions by both staff and patients provided both a comprehensive pathway of care for assessing and monitoring the physical health of newly admitted patients and a tool for the communication of individualised risk to support shared decision-making. Furthermore, creating interventions to support patients post-discharge ensured continuity of support, maximising the likelihood of sustained behaviour change, risk reduction strategies and self-care via the Recovery College model [24].

### Understanding and working in complex systems

The diversity and range of QI approaches used within the initiative gave multiple opportunities to gain insight into the problem at the clinical and organisational levels. Furthermore, they offered a framework to support the development and exploration of potential solutions that could be tested and scaled up. This was supported by the collection and reporting of improvement measures to demonstrate the successes of the initiative to the improvement team and the wider clinical staff. The improvement team attended quarterly collaborative learning events, hosted by CLAHRC Northwest London, where they further developed skills and experience in the QI methods, and had the opportunity to test the dissemination of their findings.

Although the initiative was developed on a single ward it used a systems approach allowing links across and within the organisation to be identified with the explicit aim of extending beyond the immediate setting to services provided by the wider organisation. At the micro/clinical level the process mapping was used to explore local systems and processes which identified duplication of activities that were subsequently targeted for improvement. The design of the interventions was specifically supported using PDSA cycles, which encourage rapid tests of change to iteratively develop de novo interventions within their implementation context. Whilst the concept was embraced by the improvement team, the reality of structuring and recording high fidelity cycles was seen to be much harder. This meant that on occasions the concept was retrospectively applied to some activities. For example, the assessment form was iteratively developed, but no contemporaneous record was made of who it was tested on, when, how and what the results were. Similarly, the challenges associated with the use of ‘measurement for improvement’ as a QI approach were not related to the lack of data collection and analysis, but a difficulty in initiating ‘action’ due to signals in the data.

### Engaging with staff and patients

Service user involvement within the initiative was critical to the success through the development of the patient-held record and in establishing an ethos for improvement: one of candour and collaboration. The 4PI framework, developed for involving patients, was supportive in creating an improvement team with flattened hierarchies and set a tone for inclusivity and a culture of shared learning emphasising that all contributions are of equal value [14]. However, the team reflected that it was the active involvement of service users and front line clinical staff in co-producing the interventions that was generally seen as significant added value to the initiative, as involving those delivering or using services was a key aspect [31].

The availability of dedicated resources, especially a project manager without direct clinical responsibilities to oversee the day to day project management tasks and co-ordinate activities, was essential in preventing fatigue within the improvement team and ensuring regular progress meetings were held. The high-level commitment from ward staff has been achieved through regular opportunities to attend these meetings and provide input into the initiative. The ward manager and staff nurse, as ward champions for the initiative, have had key roles in brokering this link.

Specific QI methods and approaches had a role in facilitating dialogue between different stakeholders, especially those outside the immediate improvement team. The AED ensured consensus on the aim of the initiative was achieved and gave a voice to stakeholders, especially in identifying and prioritising potential solutions. Sustainability, as a concept, was recognised as important to the improvement team, and the efforts made to embed the process changes into daily routines.

### Limitations

The study has several limitations as expected with quasi-experimental studies, which by their very nature are unable to determine causal links between interventions and outcomes [32]. This study focuses on the improvement of processes, and whilst there maybe unrecognised confounders responsible for the observed changes, this does not pose a significant limitation to the study and the ability to determine the success of the QI initiative. Additionally, the report authors are those that comprised the improvement team and undertook the QI initiative delivered by the ward staff. Whilst there were no independent mechanisms for collecting data from ward staff about their perspectives of the initiative, there have been opportunities for the improvement team to reflect, captured by an embedded researcher and QI advisor (SG). The account describes the initiative and its success during the implementation period, however, its sustainability beyond the funding period has not been assessed, nor the ability to scale up and roll-out both within and beyond the current clinical and organisational setting.

## Conclusion

People with SMI have a reduced life expectancy which is largely due to the high prevalence of cardiovascular, respiratory and endocrine disorders, which in turn are often dependent on modifiable factors. Whilst public health policy and clinical guidelines have advocated prioritising the physical health of these populations, the application of these has been somewhat limited. Introducing changes to the way people with SMI have their physical health assessed and risk communicated whilst in an acute mental health setting offers an opportunity to stratify patients by risk, signpost to effective interventions and empower patients to engage with preventative services and self-care.

QI offers a pragmatic and scientific approach to exploring system barriers to physical health monitoring and builds capability and capacity for change, providing frameworks for introducing process changes that support the creation of a wider healthcare system that recognises and manages physical health as well as mental health.

As demonstrated, the initiative increased the proportion of patients that had key physical health parameters such as BMI and blood pressure assessed during admission improving the assessment of physical health related risk and its meaningful communication to patients. These are necessary steps to improve health outcomes for people with SMI and can be achieved with a QI informed collaborative approach between patients, organisational leaders and clinicians.

## Abbreviations

AEM: action and effect method
AED: action effect diagram
BMI: body mass index
CVD: cardiovascular disease
CQC: care quality commission
CLAHRC NWL: Collaboration for Leadership in Applied Health Research and Care Northwest London
NIHR: National Institute of Health Research
NAS: National Audit of Schizophrenia
NICE: National Institute of Health and Care Excellence
PHA: physical health assessment
PDSA: Plan-Do-Study-Act
QI: quality improvement
SMI: serious mental illness
SQUIRE: Standards for Quality Improvement Reporting Excellence
SPC: statistical process control
WISH: Web Improvement Support for Healthcare
